# Case report: Sulfasalazine-induced hypersensitivity

**DOI:** 10.3389/fmed.2023.1140339

**Published:** 2023-05-24

**Authors:** Ekaterina M. Kuchinskaya, Irina A. Chikova, Mikhail M. Kostik

**Affiliations:** ^1^Laboratory of Autoimmune and Autoinflammatory Diseases, Almazov National Medical Research Centre, Saint-Petersburg, Russia; ^2^Hospital Pediatry, Saint-Petersburg State Pediatric Medical University, Saint-Petersburg, Russia

**Keywords:** drug-induced hypersensitivity syndrome, DIHS, drug reaction with eosinophilia and systemic symptoms, dress, sulfasalazine, serious adverse events

## Abstract

Drug-induced hypersensitivity syndrome (DiHS)/drug reaction with eosinophilia and systemic symptoms (DRESS) is a systemic inflammatory condition that is characterized by multisystemic involvement (liver, blood, and skin), heterogeneous manifestations (fever, rash, lymphadenopathy, and eosinophilia), and an unpredictable course; cases of DiHS/DRESS caused by sulfasalazine are rare in children compared to adults. We report a case of a 12-year-old girl with juvenile idiopathic arthritis (JIA) and sulfasalazine-related hypersensitivity who developed fever, rash, blood abnormalities, and hepatitis complicated with hypocoagulation. The treatment with intravenous and then oral glucocorticosteroids was effective. We also reviewed 15 cases (67% male patients) of childhood-onset sulfasalazine-related DiHS/DRESS from the MEDLINE/PubMed and Scopus online databases. All reviewed cases had a fever, lymphadenopathy, and liver involvement. Eosinophilia was reported in 60% of patients. All patients were treated with systemic corticosteroids, and one patient required emergency liver transplantation. Two patients (13%) died. A total of 40.0% of patients satisfied RegiSCAR definite criteria, 53.3% were probable, and 80.0% satisfied Bocquet's criteria. Only 13.3% satisfied typical and 20.0% atypical DIHS criteria from the Japanese group. Pediatric rheumatologists should be aware of DiHS/DRESS due to its similarities to other systemic inflammatory syndromes (especially systemic JIA, macrophage activation syndrome, and secondary hemophagocytic lymphohistiocytosis). Further studies of DiHS/DRESS syndrome in children are needed to improve its recognition and differential diagnostic and therapeutic options.

## Introduction

Drug-induced hypersensitivity syndrome (DiHS)/drug reaction with eosinophilia and systemic symptoms (DRESS) is a rare drug reaction characterized by multisystemic involvement (liver, blood, and skin), heterogeneous manifestations (fever, rash, lymphadenopathy, and eosinophilia), and an unpredictable course (1, 2).

The pathogenesis of DiHS/DRESS remains unclear. Several characteristics in the pathogenesis have been discussed: T-cell mediated hypersensitivity, genetic predisposition (the specific HLA alleles seem to be associated with certain drug-induced cases), and the herpesvirus family (mainly HHV-6) reactivation (2–4).

The true incidence of DiHS/DRESS remains unknown as it is underdiagnosed. In children, the incidence seems to be lower than in adults (2). According to the latest reports, the most frequent causative drugs are aromatic anticonvulsants, but sulfasalazine-related cases are more rare in children than in adults (2–6).

Herein, we report a 12-year-old girl with juvenile idiopathic arthritis (JIA) and DiHS/DRESS developed after 3 weeks of starting sulfasalazine treatment. In addition, we present a literature review to evaluate the clinical features of sulfasalazine-induced DiHS/DRESS in children. We hope we can draw the attention of different physicians to clinical and laboratory features of this condition, which may help in faster diagnosis and providing better treatment.

## Case presentation

The patient is a 12-year-old girl with a recently diagnosed enthesitis-related category of juvenile idiopathic arthritis (JIA) (back pain, joint pain, mild knee effusion, heel pain, enthesitis of different locations, and MRI-confirmed synovitis of facet joints of the lumbar spine); the treatment is sulfasalazine (started at 500 mg/day, gradually increased to 1.5 g/day). On the 17th day of sulfasalazine treatment, she presented with a high fever, accompanied by a few elements of itching maculopapular rash. She was admitted to the Infection Department of our hospital as her liver function tests showed increased levels of alanine aminotransferase and aspartate aminotransferase (150 IU/L and 139 IU/L, respectively), and a viral infection was suspected.

Physical examination revealed high fever (up to 39°C), mild maculopapular eruption involving trunk and extremities, and moderate hepatomegaly. No mucosal lesions or lymphadenopathy were present. Her blood count on admission showed no abnormalities, but increased ESR (20 mm/h) and CRP level (21.6 mg/dL) were detected. Plain radiography of the chest was normal. The blood, throat, and coproculture were investigated (finally, no significant pathogens could be detected), and serology results were negative for HBsAg, anti-HAV, and anti-HCV. Anti-CMV, anti-EBNA-1, and anti-VCA (IgM) were also negative. Serum PCR detected HHV-6 (no CMV or EBV).

**Figure 1:**
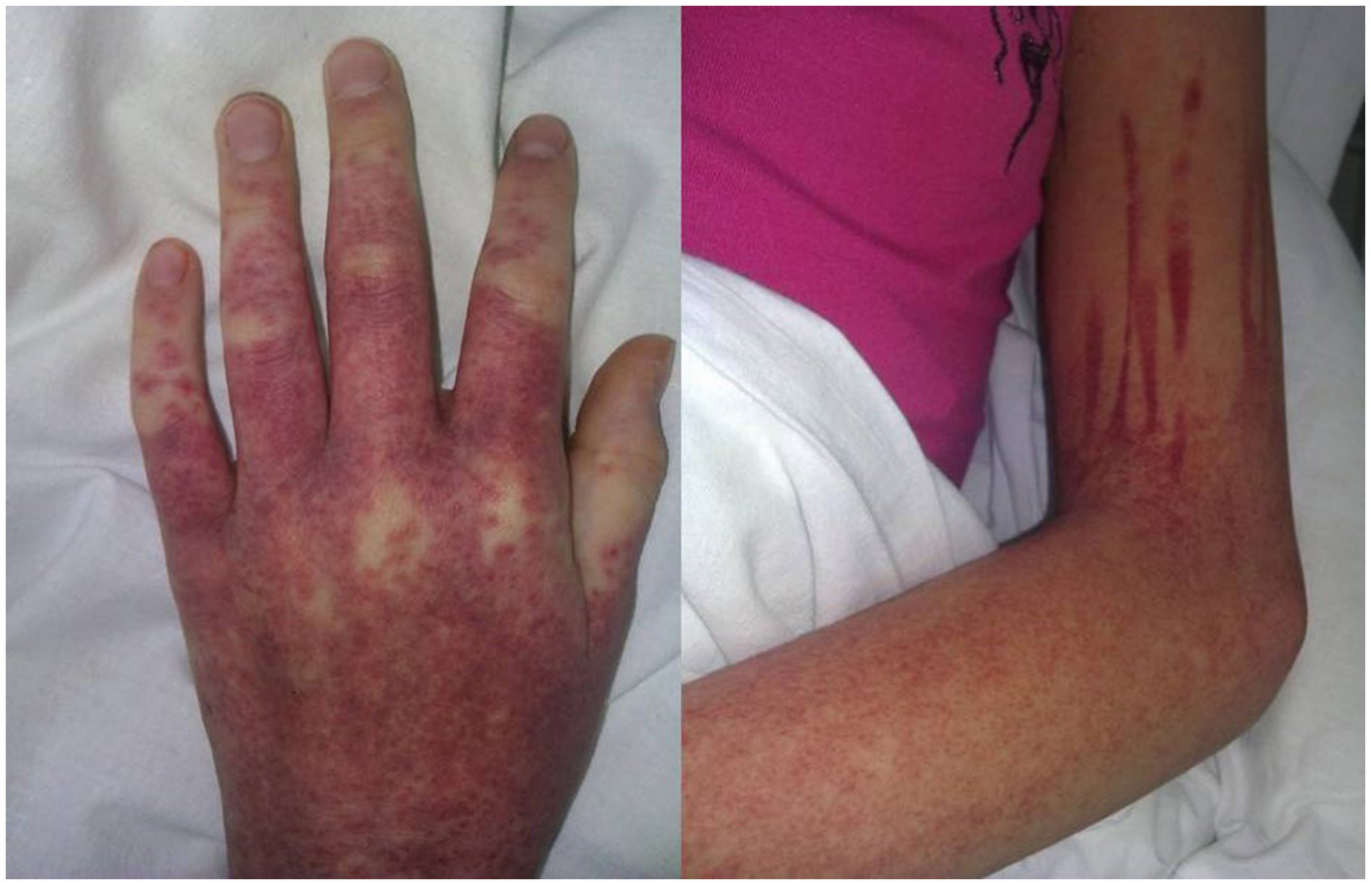
Cutaneous manifestation of DiHS/DRESS complicated by hypocoagulation: itchy maculopapular rash with hemorrhagic impregnation (Day 30 from sulfasalazine initiation).

**Figure 2:**
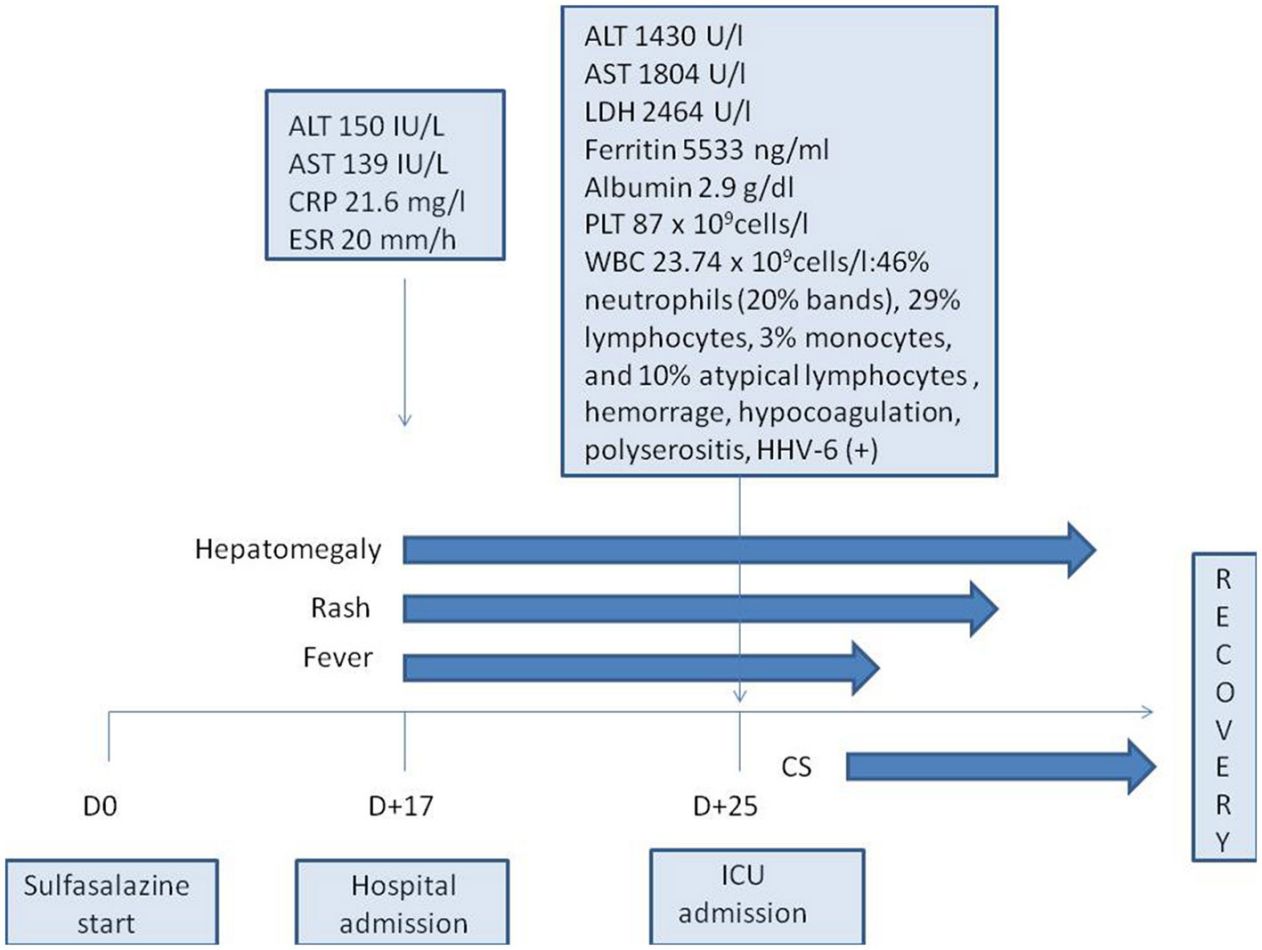
Timeline diagram of the disease course. ALT, alanine aminotransferase; AST, aspartate aminotransferase; CRP, C-reactive protein; CS, corticosteroids; D, day; ICU, intensive care unit; ESR, erythrocyte sedimentation rate; HHV-6, human herpes virus type 6; LDH, lactate dehydrogenase; PLT, platelets; WBC, white blood cells.

Sulfasalazine therapy was stopped, and empiric antibiotic therapy (ceftriaxone) was started, but the patient worsened progressively: high fever and severe fatigue persisted, and ALT and AST levels increased rapidly (up to 1,430 IU/L and 1,804 IU/L, respectively, by 8 days of admission). Increased levels of lactate dehydrogenase (2,464 IU/L, n.v. <450 IU/l) and ferritin (5,533 ng/ml, n.v. <140 ng/ml) were also revealed. Blood count showed thrombocytopenia (87 × 10^9^ cells/l) and leukocytosis (WBC up to 23.74 × 10^9^ cells/l) with 46% neutrophils (20% bands total), 29% lymphocytes, 3% monocytes, and 10% atypical lymphocytes in the peripheral blood smear. On the 8th day of admission, the patient developed hypocoagulation with intensive skin hemorrhagic rash (Figure 1), and decreased levels of total protein (49.7 g/L) and albumin (2.9 g/dL) were also presented; the ascites and pericardial effusion were confirmed by ultrasound. The patient was admitted to ICU, and the diagnosis of secondary hemophagocytic lymphohistiocytosis was supposed, but a drug-related hypersensitivity reaction was also suspected considering the history of sulfasalazine administration. Methylprednisolone pulse therapy (1,000 mg/daily for 3 days followed by oral methylprednisolone 1 mg/kg/day with tapering off over 5 weeks) and intravenous immunoglobulin (1g/kg—single infusion) were started. Rapid improvement of fatigue and fever was observed, and the liver function tests, coagulation tests, and blood count became normal within 2 weeks. Further JIA course was controlled with NSAIDs, and in 3 years, she developed hyperthyroidism. The timeline diagram is in Figure 2.

### Search strategy

We searched MEDLINE/PubMed and Scopus for articles published from inception to June 2022, using the search term “Drug-induced hypersensitivity syndrome” AND “sulfasalazine” OR “drug reaction with eosinophilia and systemic symptoms” AND “sulfasalazine” OR “DiHS” AND “sulfasalazine” OR “DRESS” AND “sulfasalazine” OR “hypersensitivity” AND “sulfasalazine”. A total of 878 publications were identified (194 MEDLINE, 194 PubMed, 326 Scopus, and 164 Web of Science core collection), and 596 titles and abstracts were screened by two authors independently after removing the duplicates. We excluded reviews, comments, editorials, and other study types without clinical data from individual patients; after that, cases of isolated skin involvement without any systemic symptoms were also excluded. The bibliographies of all included studies were revealed to find other eligible cases. All publications enrolled were in English (except for one in French (5). One publication (6) was unavailable but was mentioned in another source (6), so it was possible to include this case in our review.

The standard form of extracted data contained the author, initial diagnosis, sex, age, clinical symptoms, laboratory results, therapy, and follow-up (Table 1) (5–19).

**Table 1:** Drug-induced hypersensitivity syndrome/drug reaction with eosinophilia and systemic symptoms cases, published in the literature.

**#**	**References**	**Indication for sulfasalazine**	**Sex, age**	**Days to the onset**	**Rash**	**Fever**	**Lymphadenopathy**	**Eosinophils × 10^3^/μL**	**ALAT, U/L**	**Bocquet et al. criteria**	**RegiSCAR (# criteria)**	**J-SCAR**	**Therapy**	**Follow-up**
1	Queyrel et al. (5)	JIA (PsA)	F,15	15	+	+	+	0.9	17x UNL	-	Probable (4)	No	GCS	Alive
2	Ribe et al. (6)	UC	M,15	14	+	+	+	0.6	3830	-	Probable (4)	No	GCS	Died
3	Balci et al. (7)	JIA	M,15	28	+	+	+	1.8	690	+	Probable (5)	Typical	GCS	Alive
4	Boyer et al. (8)	CD	F,14	14	+	+	+	3.17	230	+	Definite (6)	No	GCS	Alive
5	Sussman et al. (9)	UC	M,16	21	+	+	+	1.7	604	+	Probable (5)	Atypical	GCS	Alive
6	Ferrero et al. (10)	IBD	M,15	7	+	+	+	↑	1484	+	Definite (6)	No	GCS	Alive
7	Calil et al. (11)	TRC	M,18	28	+	+	+	3.2	1768	+	Probable (5)	N/A	ELT	Died
8	Pinana et al. (12)	JIA (ERA)	M,11	28	+	+	+	1.35	1308	+	Definite (6)	Typical	GCS	Alive
9	Rosenbaum et al. (13)	UC	F,11	28	+	+	+	2.4	135	+	Definite (6)	No	GCS	Alive
10	De Greef et al. (14)	UC	M,14	21	+	+	ND	ND	↑	+	N/A	N/A	GCS	Alive
11	Arikoglu et al. (15)	JIA	M,17	NA	+	+	+	ND	↑	-	Probable (4)	N/A	GCS	Alive
12	Fathallah et al. (16)	psoriasis	F,10	5	+	+	+	Yes	↑	+	Probable (4)	N/A	GCS	Alive
13	Losek et al. (17)	CD	M,13	11	+	+	+	5.6	ND	+	Probable (5)	No	GCS	Alive
14	Gremse et al. (18)	CD	M,10	18	+	+	+	ND	1,128	+	Definite (6)	Atypical	GCS	Alive
15	Kanner et al. (19)	UC	F,13	19	+	+	+	ND	3,320	+	Definite (6)	Atypical	GCS	Alive
16	Our patient	JIA	F, 12	17	+	+	-	-	1,430	+	Probable (5)	Atypical	GCS	Alive

ALAT, alanine aminotransferase; CD, Crohn's disease; ELT, emergency liver transplantation; ERA, enthesitis-related arthritis; GCS, glucocorticosteroids; IBD, inflammatory bowel disease; JIA, juvenile idiopathic arthritis; N/A, not applicable; ND, no data; PsA, psoriatic arthritis; TRC, Toxoplasma retinochoroiditis; UC, ulcerative colitis; UNL, upper normal limit; ↑, elevated (without exact data). ^#^Number.

## Results

Our literature review detected 15 cases of sulfasalazine-induced hypersensitivity syndrome in children aged 10–18 years (5–19). Sulfasalazine in children was used mainly for a group of related diseases with strong male predisposal, including inflammatory bowel disease (IBD), psoriasis, and enthesitis-related arthritis (ERA). Therefore, although, generally, DRESS does not show any sex predominance, the majority of our group (10/15, 67%) were male patients, which might be related to male prevalence in the underlying diseases. A total of nine patients had different variants of IBD (Crohn's disease, ulcerative colitis, or unspecified colitis), and four had different JIA categories.

The time from SSZ initiation to DRESS onset is usually reported as 2–8 weeks. Our case was typical of “3-week sulfasalazine syndrome,” which is the name that existed in the literature in the previous years (20). Surprisingly, two patients (cases 6 and 12) manifested symptoms early (on day 7 and day 5, respectively).

Clinical and laboratory data were also variable. The rash might be the first manifestation of DRESS (case 6) or occur after several days of high-grade fever (case 5). In all the cases (including our one), the rash was characterized as widespread or generalized, maculopapular and pruritic, sometimes combined with periorbital swelling or facial edema. The hemorrhagic skin syndrome was described only once (case 13) as a petechial rash localized over the wrists and hands. Our patient, on the other hand, developed a generalized hemorrhagic rash due to distinct hypocoagulation and thrombocytopenia.

Eosinophilia is supposed to be a common manifestation of DRESS and one of its diagnostic criteria, but criteria sets require different eosinophil counts (>1.5 × 10^9^/L for Bocquet's and J-SCAR, >10% or 700/μL for RegiSCAR criteria set) (2–4). Only nine of 15 (60%) cases reported the eosinophil count, and it was higher than 1.5 × 10^9^/L in six and higher than 700/μL in eight DRESS patients. In some cases, eosinophilia was correlated with high WBC count (i.e., case 4 presented with 53 × 10^9^/L WBC, and 6% eosinophils provided high absolute count), and others showed low absolute count but a high proportion due to lack of leukocytosis (case 1). Our patient had up to 1.43 × 10^9^/L eosinophils (>1,000 cells in 1 μl but only 5%), and it was not enough for Bocquet's criteria (however, her blood smear revealed atypical lymphocytosis like the other five cases in our review). Thrombocytopenia (included in RegiSCAR criteria) was also presented in our case, and it was a relatively rare manifestation in literature (4 of 15 cases).

In all reviewed cases (100%), including our case manifested with hepatitis, no other internal organ involvement was described. The ALT level varied from 135 U/l (case 9) to 3,830 U/l (case 2). Hepatic damage was mainly reversible, but one patient required emergency liver transplantation (case 6; the patient died of sepsis in the postoperative period) and another one died of hepatic failure (case 2; published in 1986). Lymphadenopathy was also mentioned in all the cases (100%) except for case 10 (no data available). All the patients were treated with oral and/or intravenous glucocorticosteroids; the dosage regimen and course duration varied: The shortest one was 48 h (case 14), and the longest one was 6 months (case 1). One patient relapsed after 2 months of GCS tapering (case 2). Two patients (13%)—cases 2 and 7—died.

## Discussion

The problem of the true incidence of DiHS/DRESS is largely the problem of its terms, definitions, and diagnostic criteria. The toxic and hypersensitive conditions, which were caused by sulfasalazine, seem to be the broad and vague spectrum, including various combinations of symptoms and laboratory findings. In earlier sources, some variants of this condition can be described under the name of angioimmunoblastic lymphadenopathy (AIL) (21) or drug-induced pseudolymphoma (2). During the investigation, we detected descriptions of sulfasalazine-associated Stevens–Johnson syndrome and toxic epidermal necrolysis with the signs of hepatitis, multiple cases of skin eruption and fever without blood or hepatic abnormalities, and also one case of hepatitis with fever, thrombocytopenia, and erythroid hypoplasia without any cutaneous manifestations (22–27).

In clinical practice, it is difficult to differentiate hypersensitivity from a direct toxic reaction, as hepatotoxicity and blood dyscrasias are well-known sulfasalazine-related adverse effects (28). There are no universal criteria for these diseases, and the following sets of diagnostic criteria are proposed: Bocquet's criteria (2), RegiSCAR (The European Registry of Severe Cutaneous Adverse Reactions to Drugs and Collection of Biological Samples) criteria (3), and SCAR-J (Japanese group of Severe Cutaneous Adverse Reactions to Drugs) criteria (20) (Table 2). Bocquet's set seems to be the most relevant one for clinical practice as it requires only three matches: drug eruption, blood changes (eosinophilia >1.5 × 10^9^/L or atypical lymphocytosis), and any organ involvement (including lymphadenopathy). Of 15 reviewed cases, 12 met Bocquet's criteria. It does not require the use of rare (e.g., thrombocytopenia), retrospective (e.g., prolonged clinical symptoms), or disputable (e.g., human herpesvirus-6 reactivation) criteria such as RegiSCAR and SCAR-J sets, but it is strongly recommended to use all sets of criteria simultaneously. Fever, being an important and stable symptom of DRESS, is not included in Bocquet's criteria, and blood changes are extremely variable and not limited by eosinophilia and atypical lymphocytosis only. For the RegiSCAR criteria, the scoring system was proposed (3).

**Table 2:** Three proposed diagnostic criteria of DRESS syndrome [adopted from Mori et al. (1)].

	**Bocquet et al. (2)**	**RegiSCAR (3)**	**J-SCAR (4)**
Requirement for diagnosis	≥3 criteria	Scoring system: definite (score >5), probable (score 4–5), possible (score 2–3) and no (score < 2)	All 7 criteria = typical without 2 asterisk marks = atypical^#^ lymphadenopathies were determined by physical examination or computed tomography
History			1) Symptoms persisting at least 2 weeks after drug discontinuation
Fever		0 points, No/Uknown −1 point	2) Fever ≥38°C
Cutaneous finding	1) Skin eruption	2) Skin rash extent >50% +1 point; 3) At least two of the following: edema, infiltration, purpura, scaling: +1 point, Unknown = 0 points, No = −1 point 4) Biopsy suggesting DRESS: +1 point, Unknown = 0 points, No = −1 point	3) Macular rash developing 3 weeks after starting offending drug
Hematologic abnormalities	2) Eosinophilia (>1.5 × 10^3^/μL) *or* atypical lymphocytosis	3) Eosinophilia: 700–1,499//μL (10–19.9%^*^) +1 point, ≥1,500 (≥20%^*^) +2 points; ^*^if leucocytes < 4,000 //μL 4) Atypical lymphocytes +1 point	4) One of the following hematologic abnormalities - leucocytosis (>11 × 10^3^/μL) - atypical lymphocytes (>5%) - eosinophilia (>1.5 × 10^3^/μL)
Other organ involvements	3) Lymphadenopathy ≥2 cm in diameter *or*- hepatitis with liver transaminases ≥2 times the normal values *or*- interstitial nephritis *or*- interstitial pneumonitis *or*- carditis	6) Lymphadenopathy = 1 point 7) One internal organ involvement +1 point; Two or more internal organ involvement +2 points.	5) Lymphadenopathy^#*^ 6) Liver abnormalities (ALT >100 U/L) or involvement of other organs
Viral reactivation			7) HHV-6 reactivation^*^
		Resolution in >15 days, No −1 point, Yes 0 point.	
		Alternative diagnoses excluded (by ≥3 biological investigations) Yes +1 point, Unknown 0 point.	

^#^Number. ^*^Marks criteria necessary for diagnosis.

A total of 40.0% of the patients satisfied the RegiSCAR definite criteria and 53.3% were probable, and 80.0% satisfied Bocquet's criteria. Only 13.3% satisfied typical and 20.0% atypical DIHS criteria from the Japanese group. Of a total of 40.0% of patients who fit the RegiSCAR definite criteria, 100.0% also satisfied Bocquet's criteria; reciprocally, 80.0% of patients who met Bocquet's criteria also satisfied the RegiSCAR definite criteria. Our patient met Bocquet's criteria, probable RegiSCAR (5/7 points), and atypical J-SCAR (6/7 points) criteria. Naranjo's algorithm evaluated the reaction to sulfasalazine as a probable adverse drug reaction The WHO-UMC system evaluated this reaction as possible. A multidisciplinary approach with a dermatologist, an immunologist, an allergologist, a clinical pharmacologist, a gastroenterologist, and other physicians is required for prompt and correct diagnosis and treatment.

Practicing physicians should keep in mind that sulfasalazine hypersensitivity might be associated with sulfonamide allergy and, therefore, with the sulfapyridine moiety of the drug (1). It is necessary to avoid the use of sulfamethoxazole–trimethoprim, salazopyrine, dapsone, and sulfadiazine if the patient had the DRESS syndrome when taking one of these drugs and warn patients about it.

## Conclusion

Pediatric rheumatologists should be aware of DiHS/DRESS due to its similarities to other systemic inflammatory syndromes (especially systemic JIA, macrophage activation syndrome, and secondary hemophagocytic lymphohistiocytosis). These conditions share such clinical features as fever, rash, lymphadenopathy, internal organ involvement, and cytopenia (or hyperleukocytosis in the case of sJIA). Moreover, DiHS/DRESS can be caused not only by sulfasalazine but also by naproxen, trimethoprim-sulfamethoxazole, infliximab, canakinumab, and anakinra. These drugs are often used in rheumatic diseases (1). Further studies of DiHS/DRESS syndrome in children are needed to improve its recognition and differential diagnostic and therapeutic options.

## Data availability statement

The original contributions presented in the study are included in the article/supplementary material, further inquiries can be directed to the corresponding author.

## Ethics statement

Ethical review and approval was not required for the study on human participants in accordance with the local legislation and institutional requirements. Written informed consent to participate in this study was provided by the participants' legal guardian/next of kin. Written informed consent was obtained from the participant/patient(s) for the publication of this case report.

## Author contributions

All authors were involved in the conception, drafting, and critical revision of the article. All authors have read and approved the final manuscript.
